# A Systematic Review of Baseline Characteristics of Patients With Successful Decongestion on Combination Diuretic Therapy in Acute Decompensated Heart Failure (ADHF)

**DOI:** 10.7759/cureus.76697

**Published:** 2024-12-31

**Authors:** Mounika Nagarani Tunuguntla, Pranathi Chanti, Sanjana S Nelogal, Midhuna Maddala, Deepa Polinati, Rukhaya LNU, Afrasayab Khan, Sweta Sahu, Salma Younas

**Affiliations:** 1 Internal Medicine, Guntur Medical College, Guntur, IND; 2 Internal Medicine, Osmania Medical College, Hyderabad, IND; 3 Internal Medicine, Salmani Hospital, Ranebennur, IND; 4 Medicine, Kakatiya Medical College, Warangal, IND; 5 Internal Medicine, Fortis Hospital, Bangalore, IND; 6 Internal Medicine, Dr. VRK Women’s Medical College, Hyderabad, IND; 7 Internal Medicine, Central Michigan University College of Medicine, Saginaw, USA; 8 Internal Medicine, JJM Medical College, Davanagere, IND; 9 Pharmacy, Punjab University College of Pharmacy, Lahore, PAK

**Keywords:** acute decompensated heart failure, baseline characteristics, combination therapy, decongestion, diuretic therapy, loop diuretics, thiazide diuretics

## Abstract

Acute decompensated heart failure (ADHF) often necessitates combination diuretic therapy when patients develop resistance to loop diuretics alone. Understanding baseline characteristics predictive of successful decongestion is essential for tailoring treatment strategies. This systematic review aimed to identify baseline characteristics associated with successful decongestion in ADHF patients undergoing combination diuretic therapy and to evaluate the effectiveness and risks of these therapies. A systematic review was conducted following Preferred Reporting Items for Systematic Reviews and Meta-Analyses (PRISMA) guidelines, with a literature search spanning 2019 to 2024 across PubMed and other databases. Studies included randomized controlled trials (RCTs) and clinical trials focusing on combination diuretic therapy, including thiazide diuretics and acetazolamide. Inclusion criteria emphasized studies reporting baseline characteristics and decongestion outcomes.

From 43 initially identified articles, four studies met inclusion criteria. Combination diuretic therapy improved diuretic response and congestion resolution, with specific therapies such as metolazone, hydrochlorothiazide, and tolvaptan showing varying levels of effectiveness. Patients with lower baseline congestion scores, preserved renal function (estimated glomerular filtration rate (eGFR) ≥41 ± 20 mL/min/1.73m²), and higher ejection fractions (≥35%) were more likely to experience favorable outcomes. Risks such as renal impairment occurred more frequently in patients with pre-existing chronic kidney disease (CKD), highlighting the importance of careful monitoring. Notably, metolazone led to greater weight loss (e.g., −6 kg vs. −3 kg) and enhanced diuretic response (940 ± 149 mL/40 mg furosemide/day vs. 541 ± 314 mL). Combination diuretic therapy is effective for improving fluid management in resistant ADHF, but baseline patient characteristics significantly influence outcomes. This review provides novel insights into the role of individualized treatment strategies in optimizing therapy. Future research should focus on validating biomarkers, risk stratification tools, and promising but understudied combinations to improve safety and efficacy.

## Introduction and background

Acute decompensated heart failure (ADHF) is a complex clinical syndrome characterized by the sudden exacerbation of chronic heart failure symptoms, including shortness of breath, fatigue, and fluid retention. This condition arises from cardiac dysfunction, resulting in pulmonary and systemic congestion, hypoperfusion, or a combination of both, significantly impacting morbidity and mortality worldwide [1]. ADHF involves both backward and forward failures. Backward failure, caused by left ventricular dysfunction due to volume or pressure overload, leads to symptoms such as dyspnea. In contrast, forward failure, stemming from reduced peripheral circulation, results in renal dysfunction, shock, and cardiac cachexia [2].

Several precipitating factors contribute to ADHF episodes, including respiratory infections, dietary transgressions such as fluid intake exceeding 2.5 liters daily or excessive salt consumption, non-compliance with medications, arrhythmias such as atrial fibrillation (AF) with rapid ventricular response (RVR), cardiac ischemia, hypertensive crisis, anemia, and worsening renal function [1]. For example, AF with RVR is frequently observed in ADHF and is associated with significant prognostic implications due to its impact on hemodynamics and cardiac output [3]. Similarly, dietary transgressions disrupt fluid and sodium balance, precipitating acute congestion, particularly in vulnerable patients [4].

Globally, chronic heart failure (CHF) affects 64.34 million people, contributing to 9.91 million years lost due to disability (YLDs) and incurring an estimated $346.17 billion in healthcare costs annually [5]. While prevalence and healthcare burden vary by region, with higher rates observed in low- and middle-income countries due to limited access to advanced therapies, ADHF poses a universally significant challenge [4]. Beyond the financial burden, ADHF is associated with high hospitalization rates and significant mortality, underscoring the need for innovative therapeutic strategies. Despite its global prevalence and devastating clinical course, ADHF lacks new approved therapies, leaving many patients reliant on traditional management approaches that often fail to address persistent congestion effectively [4]. Recent research has highlighted gaps in treatment, particularly for patients with refractory symptoms and diuretic resistance, where therapeutic innovation is urgently needed [5]. The absence of advancements in medical management not only perpetuates the cycle of recurrent hospitalizations and poor outcomes but also underscores the critical importance of exploring novel strategies to optimize care and improve quality of life for these patients.

Effective decongestion plays a pivotal role in the management of ADHF by influencing several critical clinical outcomes. Alleviating congestion significantly reduces symptoms such as dyspnea, orthopnea, and edema, thereby enhancing patients' quality of life and functional status. Hemodynamic stabilization is another crucial benefit, as reducing fluid overload decreases filling pressures in the heart, improving cardiac output and lowering the heart's workload. Furthermore, effective decongestion positively impacts the cardiorenal syndrome by reducing renal venous pressure, which in turn improves renal perfusion and function, mitigating the adverse interactions between heart and kidney dysfunctions. Achieving and maintaining decongestion is also associated with a reduced likelihood of heart failure exacerbations, leading to fewer hospital readmissions- a key factor in lowering healthcare costs and improving patient outcomes. Finally, persistent congestion at discharge correlates with higher morbidity and mortality rates; thus, effective decongestion improves both short-term and long-term prognosis by minimizing the risk of complications and recurrent heart failure episodes [6].

Achieving adequate fluid removal is linked to reduced symptoms, lower morbidity and mortality rates, and fewer hospital readmissions. Proper decongestion alleviates symptoms, decreases the cardiac workload, and prevents further deterioration of heart function, leading to improved patient outcomes.

The primary objective in acute heart failure (AHF) management is to alleviate hypervolemia through diuretic therapy, which is essential for hospitalized patients. Many individuals who receive intravenous diuretics during hospitalization require continued loop diuretic therapy after discharge to prevent symptom recurrence and rehospitalization. Monitoring includes careful assessment of fluid balance, vital signs, daily weights, and signs of congestion and hypoperfusion. Studies indicate that approximately 80% of patients with acute decompensated heart failure require diuretics upon discharge, reflecting the ongoing burden of fluid management in this population [7]. It's crucial not to discontinue diuretics prematurely due to minor changes in kidney function. Despite receiving optimal therapy, numerous patients still need diuretics after discharge, and the impact of new therapies like angiotensin receptor-neprilysin inhibitors (ARNIs) and sodium-glucose cotransporter 2 inhibitors (SGLT2is) on post-discharge diuretic dosing remains uncertain. Vasodilators may be administered to relieve pulmonary congestion in specific patients, although their long-term benefits on outcomes are unclear [8].

**Figure 1:**
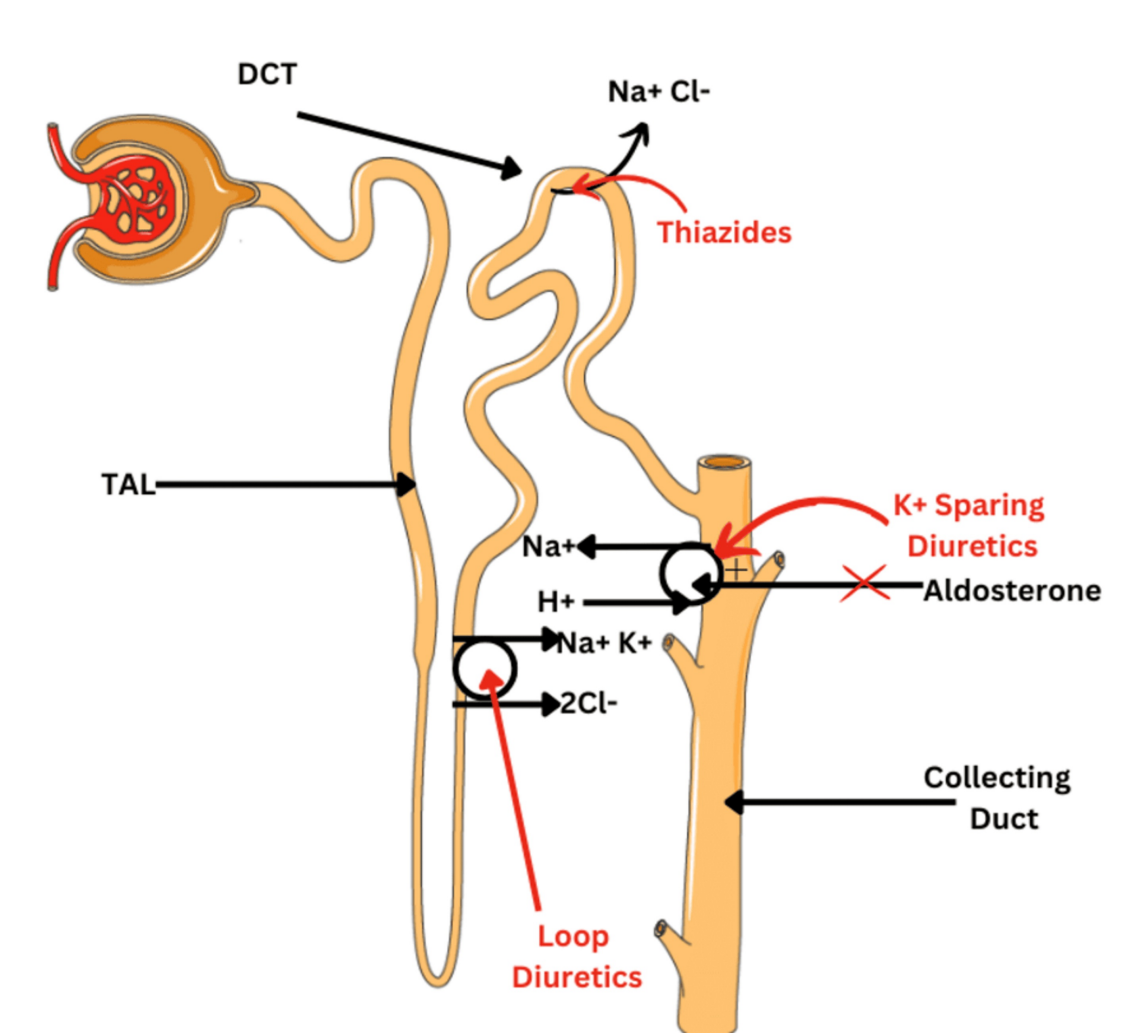
Loop diuretics function by inhibiting the Na+-K+-2CI- symporter located in the thick ascending limb cells of the loop of Henle TAL: Thick Ascending Limb, DCT: Distal Convoluted Tubule Image credits: Sweta Sahu, Mounika Nagarani Tunuguntla, Salma Younas

Loop diuretics function by inhibiting the Na+-K+-2Cl- symporter located in the thick ascending limb cells of the loop of Henle (LH). Thiazide diuretics exert their effects in the distal convoluted tubule by blocking the Na+-Cl- symporter. Mineralocorticoid receptor antagonists induce water and salt retention while increasing potassium and hydrogen ion excretion (Figure 1) [9].

Combination diuretic therapy is employed to enhance the efficacy of diuretic treatment in patients where monotherapy may be insufficient, particularly in cases of ADHF. This approach is especially relevant in ADHF patients because it addresses challenges such as diuretic resistance and facilitates rapid decongestion, critical for improving symptoms and preventing complications. Diuretic resistance, a common issue in ADHF, occurs when the body’s compensatory mechanisms limit the effectiveness of loop diuretics. Combination therapy overcomes this resistance by targeting different parts of the nephron to enhance sodium and water excretion, leading to more effective fluid removal [10].

The primary rationale behind combination diuretic therapy is to exploit the distinct mechanisms of action of different classes of diuretics, thereby increasing sodium and water excretion more effectively than monotherapy. For instance, thiazide diuretics inhibit sodium reabsorption in the distal convoluted tubule, while loop diuretics act on the sodium-potassium-chloride cotransporter in the ascending limb of the loop of Henle. This complementary mechanism amplifies the overall diuretic effect, providing superior management of conditions such as hypertension and heart failure. Moreover, combining diuretics mitigates compensatory mechanisms that often limit the effectiveness of a single diuretic. For example, using a potassium-sparing diuretic like spironolactone alongside a thiazide or loop diuretic helps counteract hypokalemia (low potassium levels) induced by these agents, improving the safety and tolerability of the treatment [11].

Enhanced compliance is another advantage of combination therapy, as it reduces the frequency and severity of side effects such as electrolyte imbalances, which are significant contributors to treatment discontinuation. Fewer side effects often translate to better adherence, improving patient outcomes [12]. Combination therapy allows for dose reduction of individual diuretics, which decreases the risk of adverse effects such as renal dysfunction while maintaining the desired therapeutic effect. For instance, studies show that combining loop diuretics with thiazide-like agents such as metolazone results in significant improvements in congestion resolution and symptom relief with fewer adverse events than high-dose monotherapy [13].

ADHF frequently necessitates intensive diuretic therapy to alleviate fluid overload, but patient responses to such treatment can differ greatly. Diuretic resistance poses a significant challenge in managing ADHF, contributing to the high rate of hospital readmissions in these patients. This condition occurs when individuals, particularly those with heart failure, experience a diminished response to diuretics, especially loop diuretics like furosemide, despite adequate dosing.

**Figure 2:**
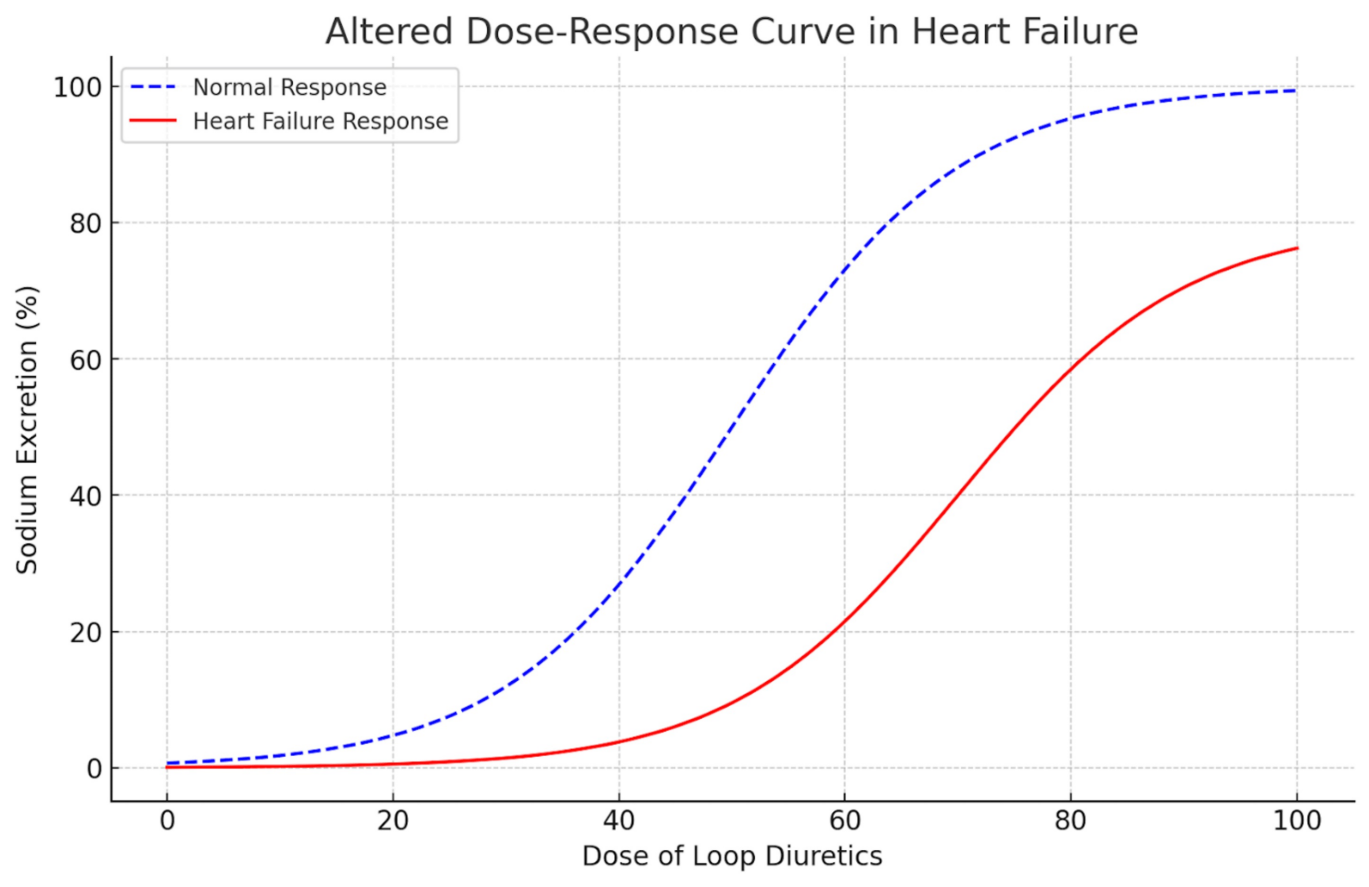
A graphical representation showing how heart failure shifts the dose-response curve for loop diuretics. The curve in red, representing the heart failure response, is shifted downward and to the right compared to the normal response (in blue). This illustrates that a higher dose is needed to achieve the same level of sodium excretion in heart failure patients. Image credits: Sweta Sahu and Mounika Nagarani Tunuguntla

Heart failure alters the dose-response curve to loop diuretics, shifting it downward and to the right (Figure 2). This implies that a greater initial dose of loop diuretics is essential to achieve the same level of sodium excretion as compared to a healthy individual. The braking phenomenon highlights the challenge of maintaining effective diuretic therapy over time, particularly in patients with heart failure, where the body’s compensatory mechanisms can limit the diuretic’s effectiveness. This phenomenon, characterized by a progressive decrease in diuretic response over time, reflects the body’s natural attempt to maintain sodium balance. In clinical practice, strategies such as sequential nephron blockade, which combines diuretics targeting multiple nephron segments, and ultrafiltration, a mechanical method of fluid removal, are employed to address this resistance and improve fluid management [10].

**Figure 3:**
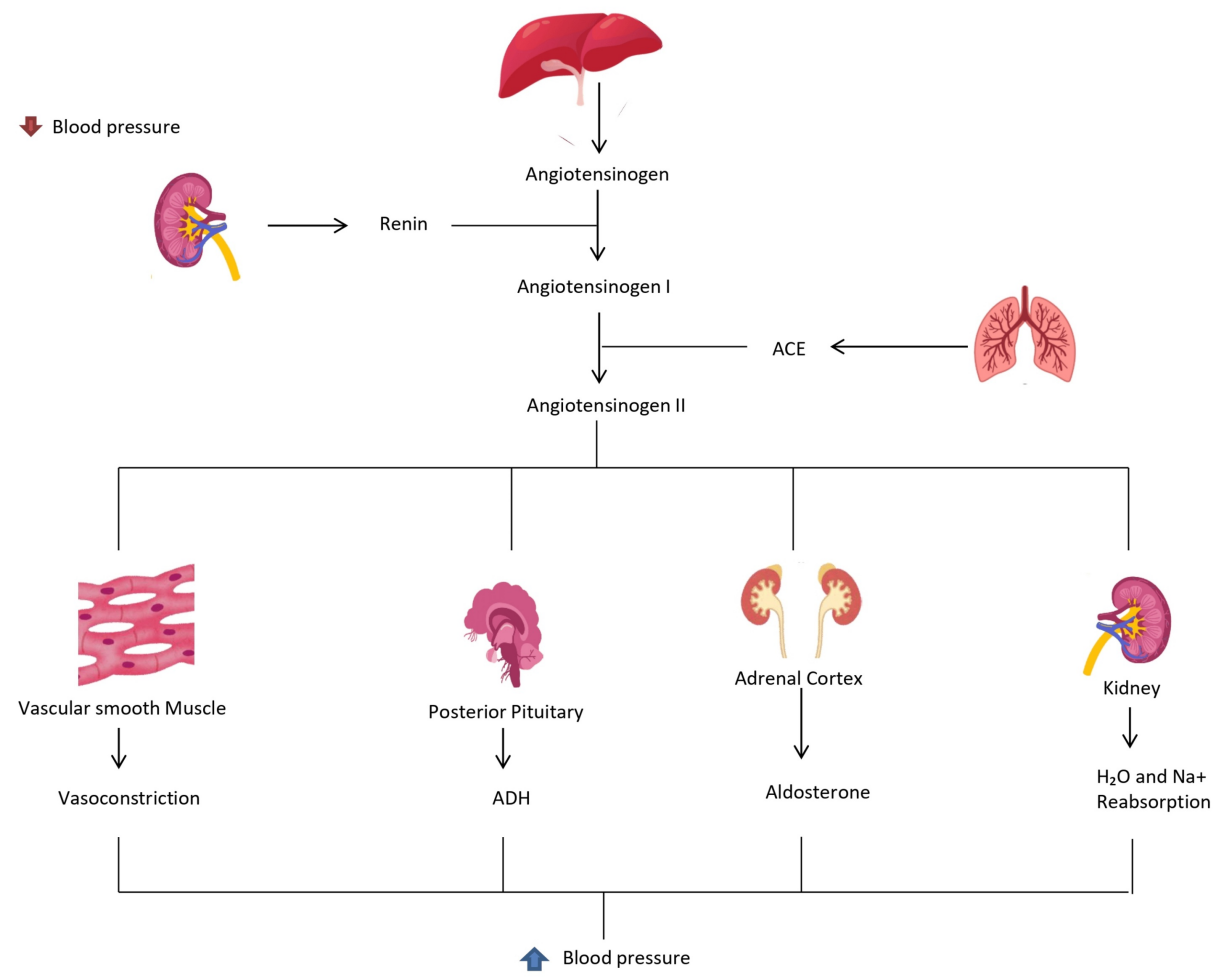
Working of the Renin-Angiotensin Aldosterone system once activated ACE: Angiotensin-Converting Enzyme, ADH: antidiuretic hormone Image credits: Mounika Nagarani Tunuguntla, Sweta Sahu, Salma Younas

This phenomenon is driven by several mechanisms-loop diuretics trigger the activation of both the renin-angiotensin-aldosterone system (RAAS) and sympathetic nervous system (SNS), which collectively reduce renal blood flow and enhance sodium reabsorption in the proximal and distal tubules (Figure 3). The decrease in renal blood flow (RBF) limits the delivery of the diuretic to its site of action within the nephron, thereby reducing its efficacy in promoting sodium excretion. This diminished delivery also results in lower concentrations of the diuretic at the loop of Henle, exacerbating the braking phenomenon and making fluid management more challenging [10]. A reduction in glomerular filtration rate (GFR) further decreases the sodium load reaching the tubules, compounding the resistance to diuretic therapy.

Continuous administration of furosemide in rats caused structural changes in the kidney, including proliferation of the distal tubule (DT), with its proportion in kidney tissue increasing significantly compared to untreated rats. This finding suggests an adaptive response to chronic loop diuretic exposure, potentially enhancing the distal tubule’s ability to actively reabsorb sodium. The study also found that cell membranes in the distal convoluted tubule (DCT) expanded, indicating an increased functional capacity for active substance transport, likely due to altered sodium levels in the tubular fluid caused by furosemide's action on the LH [14]. However, while these results provide insight into the kidney's plasticity under prolonged diuretic exposure, their translation to human physiology should be approached with caution. Differences in kidney structure and function between rats and humans may limit the applicability of these findings. Additionally, the study’s methodology, including dosage and duration of furosemide administration, and its small sample size, may not fully represent the complexity of chronic diuretic use in humans. These limitations highlight the need for further research to explore how chronic loop diuretic use affects human kidneys, particularly in heart failure patients.

The mainstay of treatment for managing fluid overload in ADHF is loop diuretics. However, prospective data for determining an optimal diuretic regimen for ADHF patients are limited. Challenges in trial design, including variability in patient responses, heterogeneity in baseline renal function, and ethical considerations for high-dose diuretic trials, contribute to this gap. As a result, current guidelines rely heavily on expert consensus rather than robust evidence. While it is well established that patients with heart failure often require higher diuretic doses, these tend to become less effective over time due to mechanisms of diuretic resistance.

An individual’s response to diuretic therapy is influenced by factors such as salt intake, diuretic dose, kidney function, and right atrial pressure [15]. Tailoring therapy to each patient’s specific needs can improve both immediate and long-term outcomes. For instance, diuretic therapy may increase sodium transport in the proximal tubule (PT), reducing the amount of sodium reaching the LH, and also enhance sodium transport in later parts of the nephron, limiting sodium excretion. These observations support the use of combination diuretics targeting different parts of the nephron to enhance efficacy.

Metolazone, with its thiazide-like activity, can be combined with furosemide therapy due to its action on the DT and ability to reduce PT reabsorption [16]. However, clinical considerations must be addressed when using this combination. Monitoring for electrolyte imbalances, such as hypokalemia, and ensuring appropriate dosing are critical to avoid adverse effects. Regular assessment of renal function and potassium levels is recommended to minimize risks associated with combination therapy while maximizing its benefits in patients with diuretic resistance.

Managing sodium intake is crucial in diuretic therapy, especially for heart failure patients. When sodium intake is high, initial sodium loss from diuretics is significant, but the kidneys quickly adjust by reducing sodium excretion. This adjustment results in little to no overall change in sodium balance or body weight because the body compensates for the loss. On a low sodium diet, even after diuretic therapy, the body struggles to restore sodium levels because the sodium loss from diuretics exceeds what is consumed through diet. This leads to a negative sodium balance, where the body is losing more sodium than it is taking in, which is crucial for reducing fluid overload in heart failure patients [17]. However, low sodium intake was associated with frequent hospitalizations and increased adverse outcomes in patients with heart failure [18].

This could be due to activation of the RAAS and SNS, leading to sodium retention and elevated potassium losses, potentially increasing the risk of death in heart failure (HF) patients. Therefore, for patients with HF with moderate symptoms and diuretic resistance, restricting sodium intake to 80 to 120 mmol (2-3 grams) daily is considered a reasonable target [19].

The CLOROTIC trial demonstrated that adding hydrochlorothiazide (HCTZ) to furosemide improved the diuretic response in acute heart failure (AHF) patients. A subsequent post-hoc analysis evaluated sex-specific outcomes in the trial. While no significant differences were observed in primary or secondary outcomes between men and women, there was a notable difference in safety outcomes, with worsening renal function occurring more frequently in women compared to men [20]. This finding, although not statistically significant, raises important questions about physiological or hormonal factors contributing to sex-specific differences in renal outcomes. For example, hormonal variations such as estrogen’s influence on renal hemodynamics and sodium handling may play a role. Difference in muscle mass and body water composition between sexes could influence pharmacokinetics and pharmacodynamics of diuretics, potentially affecting renal function. The trial’s small sample size and post-hoc nature are key limitations, which restrict the generalizability of these findings and necessitate caution when interpreting the results.

This highlights the need for careful monitoring and possibly tailored treatment approaches for different sexes to ensure safety. Through this review, we aim to identify specific patient factors (e.g., age, comorbidities, severity of heart failure, renal function) that predict a positive response to diuretic therapy in ADHF. We seek to understand how the identified baseline characteristics affect the selection and effectiveness of different diuretic combinations. It explores whether certain patient profiles respond better to specific diuretic strategies. 

## Review

Methodology

We conducted this systematic review following the Preferred Reporting Items for Systematic Reviews and Meta-Analyses (PRISMA) guidelines, as outlined in the Cochrane Handbook for Systematic Reviews of Interventions. The methodology adhered to these standards to ensure a rigorous and comprehensive evaluation of the evidence.

Search Strategy

A thorough search of the literature was performed by two independent researchers using the PubMed database. The goal was to find studies that looked at the baseline characteristics of people with ADHF who were initially not helped by loop diuretics alone, even when they were given at their highest doses. These people then needed a second diuretic, specifically thiazide or thiazide-like diuretics, to help clear out their blood vessels. The search query used in PubMed was ADHF OR acute decompensated heart failure AND (loop diuretics OR furosemide OR torsemide) AND (thiazide OR thiazide-like OR metolazone OR chlorthalidone OR HCTZ OR hydrochlorothiazide).

Table 1 summarizes the keywords and terms used in the advanced search strategy.

**Table 1:** The category and and keyword terms used in the advanced search strategy ADHF: acute decompensated heart failure, HCTZ: hydrochlorothiazide

Category	Keywords and Terms
Condition	ADHF, acute decompensated heart failure, heart failure
Loop Diuretics	sodium potassium chloride symporter inhibitors, loop diuretics, furosemide, torsemide
Thiazide Diuretics	thiazides, thiazide, thiazide-like, metolazone, chlorthalidone, hydrochlorothiazide, HCTZ
Synonyms and Variants	acutely, decompensating, decompensations, heart, failure, frusemide, furosemid, torasemide, thiazid, thiazidic, hydrochlorothiazid, thiazides
Pharmacological Action	sodium potassium chloride symporter inhibitors
MeSH Terms	sodium potassium chloride symporter inhibitors, furosemide, torsemide, thiazides, metolazone, chlorthalidone, hydrochlorothiazide

To ensure comprehensive coverage, a manual search was conducted by reviewing the reference lists of relevant publications, including prior systematic reviews and review articles. The initial screening involved assessing titles and abstracts, followed by a full-text review of potentially eligible studies. Any discrepancies in study selection were resolved by a third researcher.

Study Selection

Inclusion criteria:The study must be a randomized controlled trial (RCT) or a clinical trial. Participants should be hospitalized individuals with acute decompensated heart failure. The intervention under investigation must involve combination diuretic therapy, including thiazide or acetazolamide, compared to loop diuretics alone. The outcome of interest is the reported differences in congestion levels between patients receiving combination diuretic therapy and those receiving loop diuretics alone.

Exclusion criteria:Studies that are not RCTs or clinical trials, such as observational studies, reviews, case studies, and case series, are excluded. Additionally, studies lacking adequate data on baseline characteristics or outcomes related to congestion levels, such as missing detailed patient demographics (e.g., age, renal function, ejection fraction) or decongestion-related outcomes (e.g., weight loss, urine output, congestion scores), are excluded. This ensures that only studies providing comprehensive and interpretable data relevant to the review objectives are included, reducing ambiguity regarding "adequate data." Lastly, studies not involving the specified combination diuretic therapy (e.g., thiazide diuretics or acetazolamide) or those involving populations other than hospitalized individuals with acute decompensated heart failure are excluded.

Data Extraction

**Figure 4:**
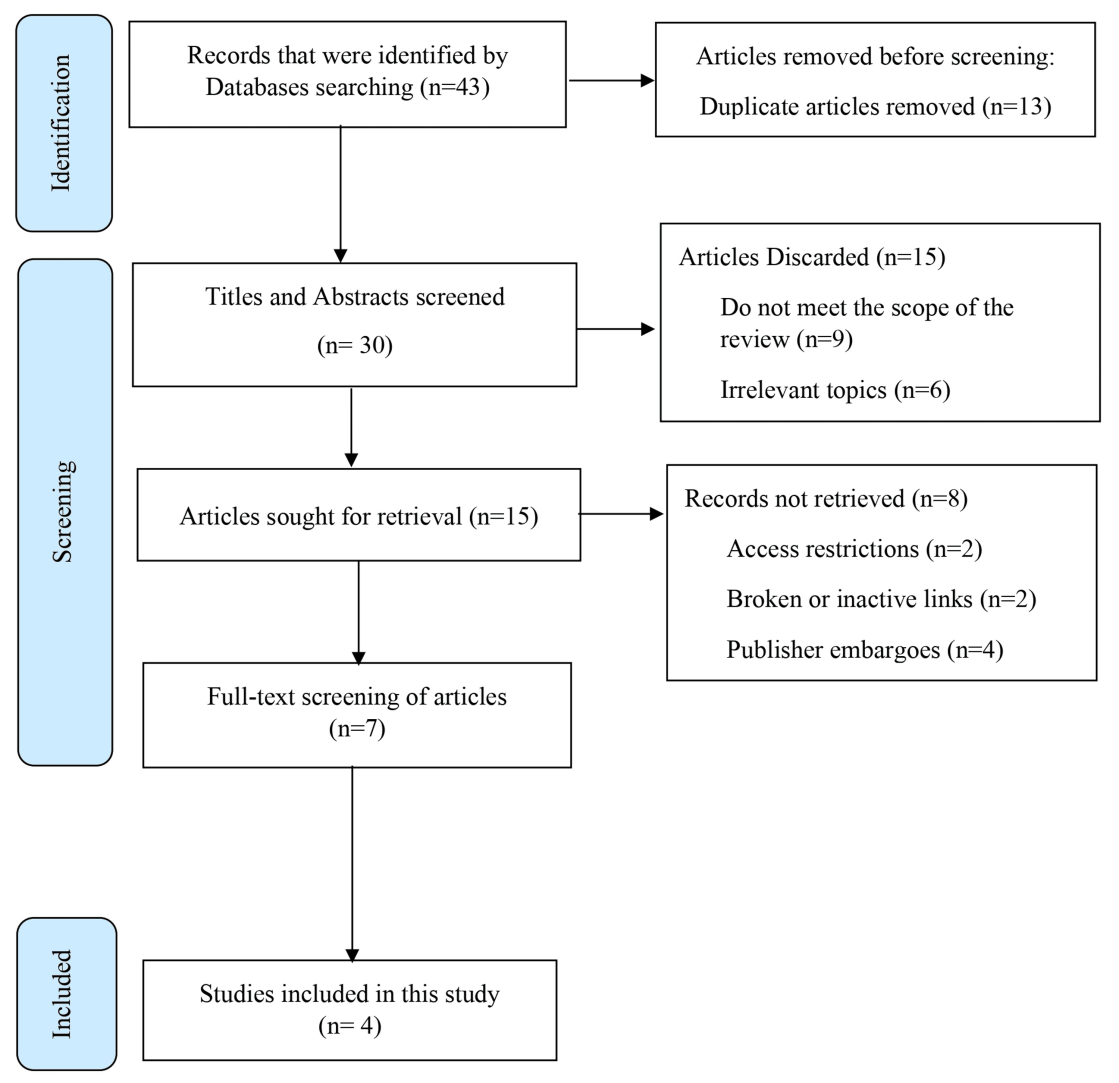
Preferred Reporting Items for Systematic Reviews and Meta-Analyses (PRISMA) flow diagram

The literature search identified 43 relevant articles. After removing duplicates, a total of 30 articles remained. Initial screening of titles and abstracts led to the exclusion of 15 articles. A further seven articles were excluded after a full-text review based on the inclusion and exclusion criteria. Discrepancies were resolved, resulting in four articles meeting the final inclusion criteria, as illustrated in the PRISMA flow diagram (Figure 4).

The final selection process focused on studies that provided comprehensive data on baseline characteristics and outcomes related to combination diuretic therapy in ADHF, ensuring that the included studies were relevant and methodologically sound (Table 2).

**Table 2:** Characteristics of the studies NT-proBNP: N-terminal pro-B-type Natriuretic Peptide, NYHA: New York Heart Association, CKD: Chronic Kidney Disease, HCTZ: Hydrochlorothiazide, IV: Intravenous, ACEIs: Angiotensin-Converting Enzyme Inhibitors, ARBs: Angiotensin II Receptor Blockers, LV: Left Ventricular

Study	Study Design	Population Characteristics	Intervention	Decongestion Outcomes	Renal Function Outcomes	Key Findings and Implications	Limitations
Palazzuoli et al. (2021) [21]	Retrospective, multicenter case-control study (n=132).	Older adults (mean age 70); NYHA class III-IV; EF < 40%; CKD prevalence: 51%-57%; high baseline loop diuretic use (230-250 mg/day).	Metolazone + high-dose furosemide vs. high-dose furosemide alone.	Improved diuretic response (940 ± 149 mL/40 mg vs. 541 ± 314 mL/40 mg; p<0.001); greater weight loss (−6 ± 2.3 kg vs. −3 ± 1.5 kg; p<0.01); reduced congestion scores (median 0.8 vs. 2; p=0.03).	No significant difference in WRF; serum creatinine stable; higher hypertonic saline use in metolazone group (33% vs. 12%; p=0.03).	Metolazone effectively enhances decongestion with minimal renal risk; NT-proBNP reduction insignificant, indicating limited systemic impact.	Retrospective design; small sample size; high-risk older population limits generalizability.
CLOROTIC Trial (2022) [22]	Multicenter, randomized, double-blind, placebo-controlled trial (n=230).	Mean age 83; 48% women; NYHA class III-IV; moderate CKD prevalent (eGFR 20-50 mL/min/1.73m²).	HCTZ + intravenous furosemide vs. placebo + intravenous furosemide.	Greater weight loss at 72 hours (−2.3 kg vs. −1.5 kg; p=0.002); 24-hour diuresis higher in HCTZ group (1775 mL vs. 1400 mL; p=0.05).	More frequent renal impairment in HCTZ group (p=0.04); no significant changes in long-term mortality or rehospitalization.	Effective for short-term decongestion; renal risk requires monitoring and dose adjustments; no significant impact on dyspnea or long-term outcomes.	Slow enrollment; imbalances in baseline characteristics; short follow-up (90 days); no post-discharge renal monitoring.
Rahimi et al. (2021) [23]	Retrospective cohort study from PROVE/HF registry (n=1438).	Mean age 68.2; 63%-72% male; LVEF < 30%; balanced comorbidities (HTN, DM, CKD).	Furosemide + metolazone (5.2 ± 2.8 mg/day) vs. furosemide alone (160.5 ± 38.8 mg/day vs. 122.4 ± 62.1 mg/day).	No significant difference in mortality (HR 0.78; p=0.085) or rehospitalization (OR 0.80; p=0.135); improved diuretic response (not quantified).	No significant differences in adverse renal outcomes; renal safety profile similar across groups.	Metolazone does not improve long-term outcomes but enhances acute decongestion; well-tolerated in routine clinical practice.	Retrospective design; single-center; lack of granular data on renal function (e.g., end-diastolic volume); no follow-up on post-discharge care.
Cox et al. (2019) [24]	Prospective, randomized, double-blind trial (n=60).	Mean age 62; 77% HFrEF; high prevalence of comorbidities (DM 70%, HTN 83%, CKD 73%); mean eGFR 41 ± 20 mL/min/1.73 m².	Metolazone (5 mg BID), IV chlorothiazide (500 mg BID), or tolvaptan (30 mg daily), all combined with high-dose furosemide.	Significant weight loss across all groups (metolazone: −4.6 ± 2.7 kg; chlorothiazide: −5.8 ± 2.7 kg; tolvaptan: −4.1 ± 3.3 kg); no significant differences between groups.	Small differences in electrolyte imbalances (tolvaptan less natriuretic but safer for sodium/chloride levels); transient creatinine changes.	No clear superiority between agents; weight loss and decongestion are similar; metolazone shows slightly higher symptomatic hypotension rates.	Small sample size; limited follow-up (48 hours); focused on diuretic-resistant patients, limiting generalizability.

Risk of Bias and Quality Assessment

The assessments were made based on the study design and potential biases, utilizing tools like the Cochrane Risk of Bias Tool for RCTs and the Newcastle-Ottawa Scale for cohort studies.

**Table 3:** Quality and risk of bias assessment using Cochrane Risk of Bias Tool and Newcastle-Ottawa Scale

Study	Study Design	Selection Bias	Performance Bias	Detection Bias	Attrition Bias
Palazzuoli et al., 2021 [17]	Retrospective Cohort Study	High risk (no randomization)	Moderate risk (no blinding)	Moderate risk (no blinding)	Low risk (no significant dropouts)
Trullàs et al., 2022 [18]	Randomized, Double-Blind, Placebo-Controlled Trial	Low risk (randomized)	Low risk (double-blind)	Low risk (blinded)	Low risk (no significant dropouts)
Cox et al., 2019 [19]	Randomized, Double-Blind	Low risk (randomized)	Low risk (double-blind)	Low risk (blinded)	Low risk (no significant dropouts)
Rahimi et al., 2021 [20]	Retrospective Cohort	High risk (no randomization)	Moderate risk (no blinding)	Moderate risk (no blinding)	Low risk (no significant dropouts)

Results and discussion

The primary aim of this systematic review was to identify baseline characteristics that predict successful decongestion in patients with ADHF undergoing combination diuretic therapy. Understanding these characteristics is critical for optimizing treatment strategies and improving patient outcomes.

The findings indicate that combination diuretic therapy effectively enhances fluid management in patients with resistance to loop diuretics alone. This therapeutic approach improves parameters such as weight loss, urine output, and congestion resolution, particularly in populations with advanced heart failure. However, the impact of these therapies on renal function and long-term outcomes varies significantly, emphasizing the need for individualized treatment strategies to balance efficacy and safety.

Study-Specific Findings

The findings of the included studies provide insights into the efficacy and safety of combination diuretic therapies in ADHF. Palazzuoli et al. demonstrated that adding metolazone to high-dose loop diuretics significantly enhanced diuretic response and congestion relief, with a mean daily diuresis of 2,820 mL and a weight loss of 6 kg compared to controls (p < 0.01) [21]. Similarly, the CLOROTIC trial highlighted that HCTZ addition led to greater weight loss (−2.3 kg vs. −1.5 kg, p = 0.002) and improved 24-hour diuresis (p = 0.05), although it was associated with a higher incidence of renal impairment [22]. In contrast, the Rahimi et al. study found no significant differences in mortality or rehospitalization with metolazone but noted an improved diuretic response (p < 0.05) [23]. The 3T Trial revealed comparable weight loss and diuretic efficiency among metolazone, IV chlorothiazide, and tolvaptan but emphasized metolazone’s superior efficacy in weight reduction [24].

Population Characteristics

The populations studied were predominantly older (mean age: 62-83 years) with significant comorbidities, including diabetes, hypertension, and chronic kidney disease (CKD). These characteristics reflect real-world ADHF populations but limit generalizability to younger or less comorbid groups. For example, in the CLOROTIC trial, 48% of participants were women, and many had moderate renal dysfunction, while the Palazzuoli study focused on advanced heart failure with reduced ejection fraction (HFrEF). The high prevalence of CKD and its impact on diuretic response is particularly notable, as patients with eGFR <30 mL/min were excluded from some studies [22].

Renal Dysfunction

Renal impairment emerged as a critical concern across studies. The CLOROTIC trial reported that HCTZ use increased the risk of worsening renal function (p = 0.04) without impacting rehospitalization or mortality [22]. For instance, the CARRESS-HF trial suggested that aggressive decongestion strategies in ADHF, including combination diuretics, could lead to worse renal outcomes without a corresponding improvement in long-term survival [25]. Moreover, the observed improvement in diuretic response with metolazone in our review is consistent with previous literature. A systematic review by Steuber et al. highlighted metolazone’s ability to potentiate the effects of loop diuretics, especially in cases of refractory heart failure [26]. In the Palazzuoli study, hypertonic saline was used to mitigate electrolyte imbalances, suggesting a potential strategy for reducing renal risks. While metolazone consistently improved fluid removal, its impact on renal outcomes was mixed, with no significant worsening in most studies [21]. These findings underscore the importance of monitoring renal function and tailoring therapies to individual risk profiles.

Clinical Implications

The lack of significant changes in N-terminal pro-B-type natriuretic peptide (NT-proBNP) levels in the Palazzuoli study suggests that improved decongestion may not correspond to systemic neurohormonal benefits [21]. This highlights a limitation of combination diuretic therapy in addressing broader pathophysiological processes. Strategies to mitigate renal dysfunction, such as cautious dose escalation, monitoring, and adjunctive therapies like hypertonic saline, could improve safety.

The Rahimi study’s findings of unchanged mortality and rehospitalization rates align with concerns from the DOSE trial, where aggressive diuretic strategies did not yield better long-term outcomes. This disconnect between improved decongestion and long-term benefits may result from the multifactorial nature of ADHF, including underlying comorbidities and disease progression. Future research should explore how combination therapy can be integrated into comprehensive management strategies, including neurohormonal blockade and sodium-glucose co-transporter-2 inhibitors [23,12].

Limitations of Included Studies

The small sample sizes and short follow-up durations in most studies limit the robustness of findings. For instance, the 3T Trial included only 60 patients and assessed outcomes over 48 hours [24]. Retrospective designs, as in the Palazzuoli and Rahimi studies, introduce selection bias, while heterogeneity in study populations and interventions complicates comparisons [21,23]. Future trials should incorporate larger, more diverse cohorts and extend follow-up periods to assess long-term safety and efficacy.

Implications for Specific Subgroups

Patients with CKD, diabetes, and HFrEF appear to benefit most from combination therapies due to their higher baseline congestion and greater diuretic resistance. However, these groups are also at higher risk for adverse renal outcomes. The CLOROTIC trial emphasized the need for tailored dosing based on eGFR, while the Rahimi study highlighted the safety of metolazone in a high-risk cohort. Stratified analyses by comorbidities and heart failure phenotypes could further refine treatment approaches.

Future Directions

Future research should focus on randomized controlled trials with larger sample sizes, longer follow-ups, and standardized protocols. Studies should evaluate the impact of combination diuretics on quality of life, adherence, and patient-reported outcomes. Investigating the interplay of comorbidities, particularly CKD and diabetes, with diuretic response could yield insights into optimizing therapy. Exploring the integration of combination diuretics with newer therapies like SGLT2 inhibitors may enhance outcomes.

## Conclusions

Baseline characteristics are key to predicting the success of combination diuretic therapy in ADHF. Patients with lower baseline congestion, preserved renal function, and higher ejection fraction achieved better outcomes, such as improved diuresis, weight loss, and congestion resolution. Therapies involving metolazone and hydrochlorothiazide were more effective than loop diuretics alone. Despite these benefits, risks like renal impairment, particularly in advanced CKD patients, highlight the need for careful monitoring. Biomarkers like NT-proBNP and clinical scoring systems can guide personalized treatment, optimizing outcomes and minimizing complications. Promising combinations like acetazolamide and loop diuretics warrant further investigation to refine treatment strategies and improve long-term outcomes.
